# Stop-Gain Mutations in PKP2 Are Associated with a Later Age of Onset of Arrhythmogenic Right Ventricular Cardiomyopathy

**DOI:** 10.1371/journal.pone.0100560

**Published:** 2014-06-26

**Authors:** Mireia Alcalde, Oscar Campuzano, Paola Berne, Pablo García-Pavía, Ada Doltra, Elena Arbelo, Georgia Sarquella-Brugada, Anna Iglesias, Luis Alonso-Pulpon, Josep Brugada, Ramon Brugada

**Affiliations:** 1 Cardiovascular Genetics Centre, University of Girona-IDIBGI, Girona, Spain; 2 Arrhythmia Section, Thorax Institute, Hospital Clinic, Barcelona, Spain; 3 Cardiomyopathy Unit, Heart Failure and Heart Transplant Section, Department of Cardiology, Hospital Universitario Puerta de Hierro, Madrid, Spain; 4 Arrhythmia Section, Hospital Sant Joan de Deu, Barcelona, Spain; University of Illinois at Chicago, United States of America

## Abstract

**Background:**

Arrhythmogenic right ventricular cardiomyopathy (ARVC) is a cardiac disease characterized by the presence of fibrofatty replacement of the right ventricular myocardium, which may cause ventricular arrhythmias and sudden cardiac death. Pathogenic mutations in several genes encoding mainly desmosomal proteins have been reported. Our aim is to perform genotype-phenotype correlations to establish the diagnostic value of genetics and to assess the role of mutation type in age-related penetrance in ARVC.

**Methods and Results:**

Thirty unrelated Spanish patients underwent a complete clinical evaluation. They all were screened for *PKP2, DSG2, DSC2, DSP, JUP* and *TMEM43* genes. A total of 70 relatives of four families were also studied. The 30 patients fulfilled definite disease diagnostic criteria. Genetic analysis revealed a pathogenic mutation in 19 patients (13 in *PKP2*, 3 in *DSG2*, 2 in *DSP*, and 1 in *DSC2)*. Nine of these mutations created a truncated protein due to the generation of a stop codon. Familial assessment revealed 28 genetic carriers among family members. Stop-gain mutations were associated to a later age of onset of ARVC, without differences in the severity of the pathology.

**Conclusions:**

Familial genetic analysis helps to identify the cause responsible for the pathology. In discrepancy with previous studies, the presence of a truncating protein does not confer a worse severity. This information could suggest that truncating proteins may be compensated by the normal allele and that missense mutations may act as poison peptides.

## Introduction

Arrhythmogenic right ventricular cardiomyopathy (ARVC) is a rare cardiac pathology (ORPHA247) characterized by a progressive myocardial fibrofatty replacement, mainly of the right ventricle (RV), although up to 50% of cases also show a left ventricular (LV) involvement [1]. This abnormality in the myocardium disrupts electrical transmission causing ventricular arrhythmias, syncope and even sudden cardiac death (SCD) [2]. The National Centre for Biotechnology Information (NCBI) establishes a wide range of prevalence of the disease (1/2500–1/5000), depending on gender (3∶1 in men) [3], and population origin. Sometimes SCD is the first symptom of the disease. In young athletes, ARVC is believed responsible for up to 15% of SCD cases [4]. Several studies have shown that around 60% of ARVC cases have a genetic origin [1], [5]. Hence, the ARVC Task Force Criteria (TFC) has recently included genetic data as part of these criteria [6].

Typically, ARVC cases have an autosomal dominant pattern of inheritance. A recessive pattern has also been described, either associated to Naxos syndrome [7] or without this Naxos phenotype [8]. ARVC is mainly caused by pathogenic mutations in genes encoding desmosomal proteins: plakophilin-2 (*PKP2*), desmoplakin (*DSP*), desmocolin-2 (*DSC2*), desmoglein-2 (*DSG2*) and plakoglobin (PG), encoded by the *JUP* gene. Up to 60% of ARVC patients carry at least one mutation in one of these genes [9]. Genetic series have reported that 30%–40% carry a pathogenic mutation in the *PKP2* gene, followed by *DSP* (10%–15%) [10], *DSG2* (3%–8%) [11] and *DSC2* (1%–5%) [12]. In addition, non-desmosomal genes have also been identified as responsible for this pathology: transmembrane protein 43 (*TMEM43),* transforming growth factor beta 3 (*TGFΒ3),* Catenin alpha-3 (*CTNNA3)* desmin (*DES*), and recently described lamin A/C (*LMNA*), titin (*TTN*) and phospholamban (*PLN*) but with a lower incidence (so far <5% all together) [13].

Genotype-phenotype studies in families affected by ARVC show an incomplete penetrance and variable expressivity [1]. It remains unclear what are the triggering factors of the ARVC phenotype in genetic carriers. These have serious implications for the patient and for family members at risk. Thus comprehensive genotype-phenotype studies are required to better understand which asymptomatic carriers are at potential risk of developing the disease. This study aims to help address this question by assessing the prevalence of known ARVC-related genes in a Spanish population.

## Methods

### Study Population

All individuals included in our study were clinically evaluated at Hospital Clinic of Barcelona (Barcelona, Spain), Hospital Puerta de Hierro (Madrid, Spain), and Hospital Sant Joan de Deu (Barcelona, Spain). The study was approved by the ethics committee of the Hospital Josep Trueta (Girona, Spain), followed the Helsinki II declaration and written informed consent was obtained from all participants.

All patients were Caucasian and native of Spain. They were identified after presenting clinical signs or symptoms of the disease. They were clinically evaluated and diagnosed according to the recently revised Task Force Criteria (TFC) of the European Society of Cardiology/International Society and Federation of Cardiology criteria for ARVC. Clinical data are shown in Table 1. Clinical evaluation of index cases and all available relatives included a complete physical examination, 12-lead electrocardiogram, 2-dimentional echocardiography, magnetic resonance imaging, exercise stress test, 24-hour Holter and genetic testing.

**Table 1:** Clinical characteristics for Task Force Criteria (TFC) score.

Index case	Age	Gender	Symptoms	I.RVsize/function		II.RVHistology		III.Repolarizationabnormalities		IVDepolarization/Conductionabnormalities		V.Arrhythmias		VI.FamilyHistory SCD		DiagnosticScore
				Maj	Min	Maj	Min	Maj	Min	Maj	Min	Maj	Min	Maj	Min	
1	50	F	Syncope	+	−	−	−	−	−	−	−	−	−	+	−	2/0
2	33	F	Palpitations	−	+	−	−	+	−	−	−	−	+	+	−	2/2
3	52	F	Syncope	+	−	−	−	+	−	−	−	−	+	+	−	3/1
4	46	F	Dizziness	+	−	−	−	+	−	−	−	−	+	+	+	3/2
5	33	M	Palpitations	+	+	−	−	+	−	+	−	+	−	−	−	4/1
6	37	F	Palpitations	+	−	−	−	−	+	+	−	+	+	+	−	4/2
7	33	M	Palpitations	+	+	−	−	+	+	−	−	+	−	+	−	4/2
8	38	F	Palpitations	+	−	−	−	+	−	−	−	+	−	+	−	4/0
9	34	M	Dizziness	+	−	−	−	+	−	−	−	+	−	−	−	3/1
10	54	F	Palpitations	−	+	−	−	−	+	−	+	−	+	−	−	0/4
11	47	M	Palpitations	+	−	−	−	−	+	−	+	−	+	−	−	1/3
12	20	M	Palpitations	+	−	−	−	−	−	+	−	+	−	−	−	2/1
13	11	M	Palpitations	+	−	−	−	+	−	−	−	+	−	+	−	4/0
14	11	M	Syncope	−	+	−	−	+	−	+	−	+	−	+	−	4/1
15	30	M	Palpitations	+	−	−	−	−	+	+	−	−	+	−	−	2/2
16	28	M	Palpitations	−	+	−	−	+	−	−	−	+	−	+	−	3/1
17	28	M	Palpitations	+	−	−	−	−	+	−	+	+	−	+	−	4/1
18	29	M	Palpitations	+	−	−	−	−	+	−	−	−	−	+	−	2/1
19	38	F	Syncope	+	−	−	−	+	−	−	−	+	−	−	−	3/1
20	24	F	Syncope	+	−	+	−	+	−	−	−	−	+	−	−	3/1
21	22	F	Sudden death	+	−	−	−	+	−	−	−	−	+	−	−	2/1
22	23	F	Syncope	+	+	−	−	+	−	−	−	+	−	+	−	4/0
23	42	M	Syncope	+	−	−	−	+	−	−	+	+	−	+	−	5/0
24	45	M	Palpitations	+	−	−	−	+	−	−	−	+	−	+	−	4/1
25	47	M	Palpitations	+	−	+	−	−	+	−	+	+	−	−	−	3/2
26	38	M	Palpitations	+	−	−	−	−	+	−	−	−	−	+	−	2/1
27	58	M	Palpitations	−	−	−	−	+	−	−	−	−	−	+	−	2/0
28	37	M	Syncope	+	−	−	−	+	−	−	+	+	−	+	−	4/2
29	19	M	Palpitations	+	−	−	−	+	−	−	−	−	+	+	−	3/1
30	41	M	Sudden death	*Post mortem diagnosis*												

### Genetic analysis

Genetic testing was performed at the Cardiovascular Genetics Centre (Girona, Spain). Genomic DNA was extracted using commercial protocols (PUREGENE DNA, QIAGEN) from blood samples. After, DNA was amplificated by polymerase chain reaction (PCR), purified by ExoSAP-IT (ISOGEN), and sequenced (Genetic Analyzer 3130XL, Applied Biosystems). The analysis of the exonic and intron-exon regions was performed by SeqScape software (SeqScape, Applied Biosystems). Patient’s DNA was screened for *PKP2* (ENST00000070846), *DSP* (ENST00000379802), *DSC2* (ENST00000280904), *DSG2* (ENST00000261590), *JUP* (ENST00000310706) and *TMEM43* (ENST00000306077).

In order to name and analyze each identified variation, and to consider their potentiatly relation with ARVC, we consulted public genetic databases (http://browser.1000genomes.org/) [14]. Identified variation were consulted in different databases to study their possible association with this pathologyARVD/C Genetic Variants Database (www.arvcdatabase.info) and Human Gene Mutation database (www.hgmd.org). However, since new exome data are questioning the pathogenicity of previously ARVC-associated genetic variants, we studied the variant frequency in general population using the Exome Sequencing Project [15], [16]. To identify potentially ARVC associated genetic variants, we selected all identified variants with a minor allele frequency lower than 1%. (MAF <0.01). All these low frequency variants and missense novel variants were accurately analyzed by Condel (CONsensus DELeteriousness score of missense SNVs data base) *in silico* platform to predict their potential pathogenicity [17]. Additionally, to analyse the potential pathogenic role of novel variants, genetic analysis was performed in 300 Spanish control subjects (600 control alleles) (non-related individuals with Spanish ancestors). To associate a novel variation with the pathology we performed a cosegregation study.

### Statistical analysis

Statistical analysis was performed using SPSS package. We analysed differences in ARVC phenotype severity using T test for independent samples: we took diagnostic score and age of the diagnosis as dependent variables, comparing groups of males and females, carriers and non-carriers and stop-gain and missense carriers. We also perfomed one-way ANOVA to analysed differences among affected genes (*PKP2*, *DSC2*, *DSG2* and *DSP*). A P value of <0.05 was considered statistically significant.

## Results

### Study population

This ARVC Spanish cohort consisted of 30 unrelated index cases who fullfilled ARVC Task Force Criteria (table 1). The average age of our cohort was 36±12 years, with only two underage patients (11 years of age both). Of the 30 index cases, 19 (65%) were male. All index cases showed involvement of the RV and had either syncope, palpitations or dizziness. There were no significant gender differences in clinical presentation (p>0.05). Additionally, 14 cases (46,7%) had a family history of SCD. Unfortunately, familial evaluation was available only in 9 of our index cases. Electrocardiograms of these 9 index cases are shown in figures S3, S4, S5, S6, S7, S8, S9, S10, and S11 in File S1. A total of 70 relatives of 9 families were also included in our study.

### ARVC related genetic variants

**Figure 1:**
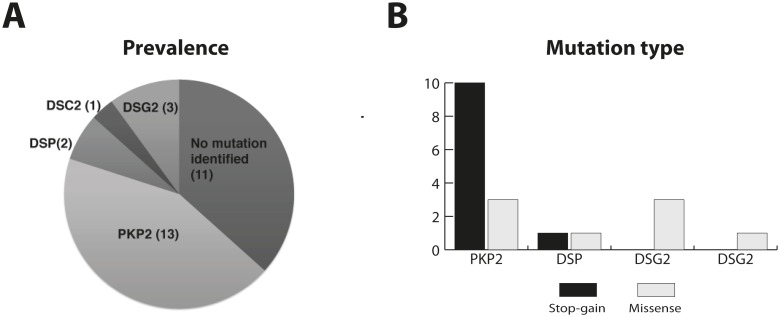
Representation of genetic results. **A**- Prevalence of mutations in desmosomal genes. **B-** Prevalence of truncating protein mutations (black) and missense mutations (grey).

We identified 17 potentially ARVC associated genetic variants in 19 of 30 index cases (63%) after screening the ARVC-related desmosome genes (figure 1A): 43% in *PKP2,* 10% in *DSC2,* 6.5% in *DSP* and 3.5% in *DSG2*. We did not find any mutation neither in *JUP* nor *TMEME43*. Eighteen patients carried the heterozygote mutations, while only one individual carried the variation (*DSG2* c.2440T>C, p.C814R) in homozygosity. There were 17 different ARVC-related variants present in 19 patients, since two individuals (13 and 28, table 2) carried the same nonsense genetic variation (*PKP2* c.275T>A, p.L92*), and two other individuals (26 and 28, table 2) carried the same deletion c.1643delG, V548fsX562 in *PKP2* gene. Of all genetic variations identified, 6 (35,3%) were novel (2 in the *DSP*, 3 in *PKP2* and 1 in *DSG2*). None of them were previously identified in genetic databases (table 2).

**Table 2:** Mutations identified in index cases.

Indexcase	Gene	Nucleotidechange	Genotye	MAF(EA)	HGMD	Predictedprotein change	*Condel*Prediction	Reportedassociationwith ARVC
1	*DSP*	c.2956C>T	HT	N/I	Novel	p.Q986*	-	-
2	*PKP2*	c.2013delC	HT	N/I	CD061457	p.P671Pfs12*	-	Pathogenic
3	*PKP2*	c.2203C>G	HT	N/I	CM043061	p.R735*	-	Pathogenic
4	*PKP2*	c.1237C>T	HT	N/I	CM060431	p.R413*	-	Pathogenic
6	*PKP2*	c.1912C>T	HT	N/I	CM043056	p.Q638*	-	Pathogenic
7	*PKP2*	c.604–605insG	HT	N/I	Novel	p.V202Vfs*13	-	-
8	*DSG2*	c.137G>A	HT	N/I	CM061701	p.R46Q	Deleterious	Pathogenic
12	*DSG2*	c.2440 T>C	HM	N/I	Novel	p.C814R	Deleterious	Pathogenic
13/27	*PKP2*	c.275T>A	HT	N/I	CM102825	p.L92*	-	-
14	*DSP*	c.6055 G>T	HT	N/I	Novel	p.A2019S	Deleterious	Pathogenic
16	*PKP2*	c. 1162 C>T	HT	N/I	CM097906	p.R388W	Deleterious	Pathogenic
17	*DSG2*	c.166G>A	HT	0.003	CM070918	p.V56M	Deleterious	Unknown significance
18	*PKP2*	c.1378 G>A	HT	N/I	^+^Cox et al 2011	p.D460N	-	Unknown significance
23	*P2*	c.2576delA	HT	N/I	Novel	p.K859Rfs*88ext*48	-	-
24	*PKP2*	c.2060 T>C	HT	N/I	Novel	p.L687P	Deleterious	-
26/28	*PKP2*	c.1643delG	HT	N/I	CD043194	p.G548Vfs*14	-	Pathogenic
29	*DSC2*	c.2194 T>G	HT	0.001	CM091021	p.L732V	Deleterious	Unknown significance

MAF = minor allele frequency consulted in European American (EA) individuals in exome sequencing project. N/I = variation not previously identified in general population. Genotype: HT = Heterozygous/HM = Homozygous. Predicted protein changed is named following Human genome variation society recommendations. ^+^Not available in public databases but already described as pathogenic mutation.

Of the 70 relatives screened, 28 were mutation carriers and 11 of them showed ARVC phenotype, positive Task Force Criteria (Table S3 in File S1) defining incomplete penetrance. None of the individuals without the genetic variation showed any symptoms or cardiac structural abnormalities related to ARVC.

### Higher relative percentatge of stop-gain mutations in *PKP2*

**Figure 2:**
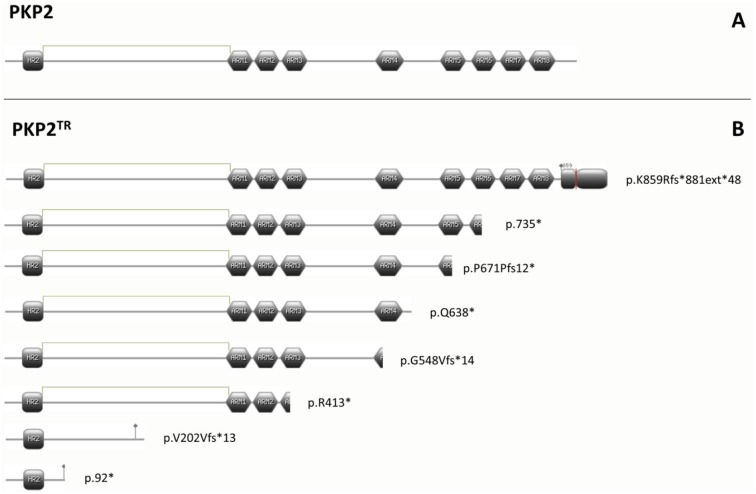
Representation of PKP2 domains. **A-** Representation of wild type PKP2. It has HR2 domain and armadillo repeats domain (8 armadillo repeats, ARM). **B-** Representation of truncated PKP2 (PKP2^TR^) and their domains. PKP2^TR^ p.92* and p.V202Vfs215* only have HR2 domain but not any of ARM repeats, p.R413* loses partially ARM 3 to C-terminus, and p.548fs562* loses partially ARM 4, p.Q638* conserve completely ARM 4 but loses the rest of protein until to C-terminus and p.R735* loses partially ARM6 to C-terminus. PKP2^TR^ p.K859V*881ent*48 extends their length to 930 amino acids.

We identified 13 individuals who carried a potentially pathogenic mutation in the *PKP2* gene. Three of them carried a missense mutation, two previously described (p.R388W and p.D460N) and one novel (c.2060T>C-p.L687P-). Variation p.D460N in PKP2 was previously reported as a genetic variant of unknown significance [5]. The remaining ten mutations were truncating PKP2 variations (PKP2^TR^). Then, PKP2^TR^ mutations represent a 52.5% of total genetically identified cases in our cohort. This PKP2 truncating group includes four *indels* -three deletion and one insertion- (c.2013delC p.P671Pfs12*, c.1643delG p.G548Vfs*14, c.604-605insG p.V202Vfs*13 and c.2576delA p.K859Rfs*881ext*48-), and four nonsense variations (c.2203C>G p.R735*, c.1912C>T p.Q638*, c.1237C>T p.R413*, c.275T>A p.L92*). Seven of these eight variations cause shorter proteins, inducing a partially or completely lost of the armadillo repeats domain (figure 2). Additionally, the variation c.2576delA p.K859Rfs*881ext*48 causes a frameshift with a final stop codon in the 3′ UTR region, and adds an extra 48 aminoacid to the protein.

In the remaining analyzed genes, we identified three missense genetic variations (10%) in the *DSG2* gene (p.R46Q, p.C814R and p.V56M). Only one of them, p.R46Q, was previously reported as pathogenic while p.V56M was classified as genetic variant of unknown significance [5]. The variation p.C814R was a novel genetic variation. Two of our 30 probands (6,7%) carried a genetic variant in the *DSP* gene (p.Q986* and p.A2019S). We found one genetic variant (3,3%) in the *DSC2* gene. The *missense* variation (p.L732V) was previously described as genetic variant of unknown significance [5]. All novel missense variations were predicted *in silico* by Condel as deleterious (table 2) and the altered aminoacid was conserved among species (figure S1 in File S1).

In summary, the relative percentatge of truncating versus missense mutations in PKP2 are significantly higher than in any other desmosomal gene (figure 1B). In fact, truncating mutations in *PKP2* gene represent 73% of the *PKP2* variations identified, while the relative percentatge is 50% in *DSP,* and zero in the remaining genes.

### Later age of onset in stop-gain mutation carriers

We identified significant differences in the age of diagnosis according to the type of mutation. Carriers with missense variations were diagnosed with ARVC at an early age (27 years old) than carriers with stop-gain mutations (39 years old) (p<0.05, T test independent samples. Table S1 in File S1). We also especifically identified a later age of onset in PKP2^TR^ than missense carriers (p<0.05, T test independent samples, excluding stop-gain in *DSP*), since all stop-gain mutations were in *PKP2*, beside one in *DSP*. Besides index cases, we also found significant differences in the age of onset when analysing 30 individuals mutation carriers already showing ARVC phenotype, fullfiling TFC (19 index cases and 11 relatives) from families; we found very similar results than in index cases, thus stop-gain carriers were diagnosed with ARVC at 38 years old and missense carriers at 27 years old (table S2 in File S1).

**Figure 3:**
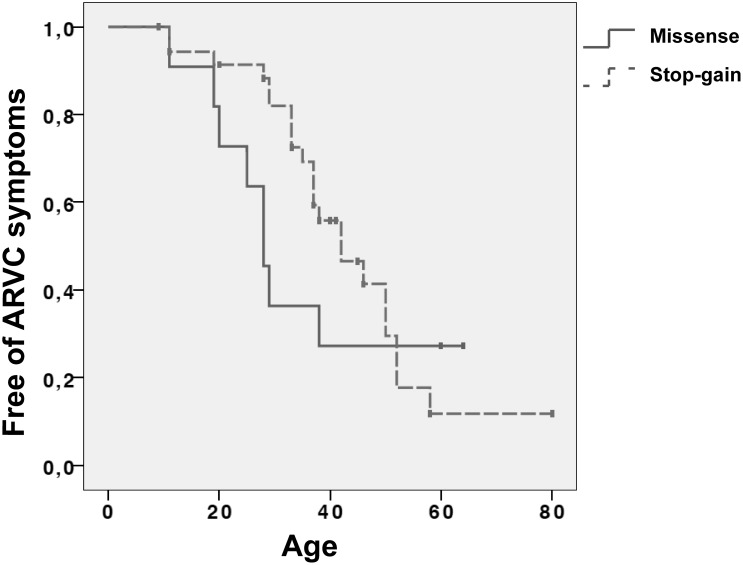
Symptom-free Kaplan-Meier graph. Kaplan–Meier graph shows percentage of free of sympthops in carriers based on age. A cross represents carriers with no ARVC phenotype by the time of the study (censored cases).

The Kaplan–Meier graph for freedom of ARVC phenotype for groups missense and stop-gain carriers was performed taking all genetic carries 47 mutation carriers (19 index cases and 28 relatives). The graph (figure 3) showed that symptom-free rate was lower in missense carriers group than missense carriers group, i.e stop–gain group has a higher percentage of free of symptoms individuals, until 50 years-old. There were 17 carriers who remain still asymptomatic with a wide range of age (9–80).

### No difference in TFC score

Task Force criteria score was not significantly different between in index cases (carriers and non-carriers, table S1 in File S1). We did not identify significant differences in the clinical manifestations of patients according to gene affected nor according to the type of mutation.

### Familial cosegregation

**Figure 4:**
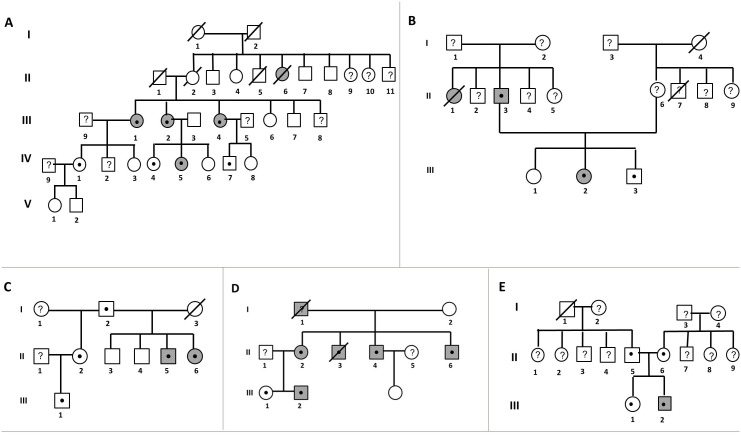
Pedigrees from 5 families. ARVC clinically affected individuals (grey round/square) and genetic carriers (black point inside round/square), not evaluated (question mark inside round/square), death (round/square with slash) and sudden death (grey round/square with slash). **A-** Family A. Index case is III.4 and carries c.2203 C>G (p.R735*) in *PKP2* ***.*** **B-** Family B. Index case is III.2 and carries c.2013delC (p.P671Pfs12*) in PKP2. **C-** Family C**.** Index case is II.4 and carries c.1912C>T (p.Q638*) in *PKP2*. **D-** Family D**.** Index case is II.1 and carries c.1237C> T (p.R413*). **E**- Family E. Index case is III.2, who carries a homozygous c.2440T>C (p.C814R) variation in the *DSG2* gene.

The 5 most relevant families, with at least three carriers in the family (table S3 in File S1), are explained in detail below. In family A (figure 4A) index case is III.4. Two out of the five siblings carry the mutation. In the third-generation we identified 3 carriers; one of them showed ARVC phenotype (IV.5), the other two (IV.4 and IV.7) did not show any clinical symptoms of the disease at an early age (27 and 20). In family B (figure 4B), the ARVC causing variation is *PKP2* c.2013delC (p.P671Pfs12*). Index case is III.2. This family showed one case of SCD (II.1) in the father’s branch. Only the index case’s father carried the variation c.2013delC (p.P671Pfs12*). This variation was identified in two relatives (II.3 and III.3), but only one of them showed an ARVC phenotype (II.3).

In family C (figure 4C), the ARVC causing variation is *PKP2* c.1912C>T (p.Q638*). Index case is II.5. Only two (II.5 I II.6) of five carriers (I.2, II.2, II.5, II.6 and III.1) showed ARVC symptoms.

In family D (figure 4D) the ARVC causing variation is *PKP2* c.1237C>T (p.R413*). Index case is II.2. In this family the identified variation showed complete penetrance, since all mutation carriers showed clinical ARVC phenotype (II.3, II.4, II.6 and III.2,) and there have even been two cases of sudden death (I.1 and II.3).

Family E (figure 4E) included evaluation of 4 members. The index case is III.2, who carries a homozygous c.2440T>C (p.C814R) variation in the *DSG2* gene. The proband showed ARVC phenotype at the age of 20 years while the heterozygous carriers (II.5, II.6 and III.1) are clinically unaffected. Electrocardiogram of index case is shown in figure S2 in File S1.

## Discussion

The present work aims to assess the prevalence of known ARVC-related genes in a Spanish population, to establish the diagnostic value of genetics and to assess the role of mutation type in age-related penetrance in ARVC.

Several data are in accordance with previous studies. Namely, average age of presentation, 36±12 years [18]; low incidence of ARVC in children (only two of our index cases were children, 11 years-old) [19], [20]; as well as similar prevalence of ARVC in men (65% in our cohort) [3], [21]. In addition, we identified a potential disease-causing variation in 63% of cases; with 40% of ARVC cases carrying a pathogenic mutation in the *PKP2* gene, supporting this as the main gene responsible for ARVC. These genetic data are also similar to previously published data on Western ARVC cohorts [22]–[25].

Compound genetic variations have been reported in some ARVC studies, representing nearly 30% of the total ARVC cases [18], [26]. In our study we did not identify any index case carrying more than one mutation, despite that we analysed all the most prevalent ARVC genes. On the other hand, we identified six novel mutations in known genes, highlighting that the investigation for only known ARVC mutations may miss some cases of genetic ARVC.

ARVC is an autosomal dominant genetic disease although there are some reported cases with a recessive pattern [8]. We identified one homozygous case, variation in the *DSG2* gene, who clearly showed an ARVC phenotype (family E), suggesting recessive pattern for this variant in this family. This fact supports that ARVC may also be present in its recessive form without associated palmoplantar keratoderma and woolly hair, in concordance to previous reported studies [27], [28]. There is always a possibility that both heterozygous carriers present a minimal form of the disease, which escapes present diagnostic technologies. The presence of double mutants in other diseases has been linked to more severe phenotypes [29].

### Type of mutation

PKP2^TR^ mutations are the most common ARVC-related genetic variations, representing 52.5% of the total ARVC variants. They are responsible for 33.5% of the total ARVC cases included in this study. These results are according with previous genetics studies in ARVC patients. In our study, all families carrying PKP2^TR^ showed incomplete penetrance, except family D carrying p.R413* where all carriers were symptomatic. This incomplete penetrance and variable expressivity in PKP2^TR^ was already described in previous studies [30].

The synthesis of PKP2 is crucial for protein interactions in myocytes, considered key point to developing the disease [31]. Likewise, nuclear localization of plakoglobin is essential for progenitor cardiac cells differentiation into adipocytes, triggering suppression of canonical Wnt/beta-catenin signaling [32], [33].

The pathogenicity of missense and truncating variations in cardiac diseases is a matter of intense debate at present, especially in structural diseases like cardiomyopathies [34]. Stop-codon mutations in PKP2 have been considered more pathogenic because they alter protein length. Truncating PKP2 proteins may lead to haploinsufficiency because of their instability [31]. This would be the most likely cause for the genesis of dominant ARVC associated with mutations in PKP2. In addition, while the process of degradation remains unclear, some previous studies have shown diminished protein levels of PKP2 in immunoblot and immunohistochemical analyses [25], [35]. Thus, the presence of truncating PKP2 mutations would confer a worse phenotype, with a symptom presentation at a younger age. This was shown in a recent study in a Japanese cohort which found that truncating PKP2 mutations are associated with the development of the disease at a significantly younger age than other mutation carriers [16]. However, in contrast with this study, our work shows that stop-gain variations in *PKP2* are associated with a later age of onset ARVC. Stop-gain carriers showed a mean age of diagnosis 36–37 years old while missense carriers have a significantly earlier age of onset (27 years old). This raises an important alternative hypothesis as to the pathogenicity of missense and truncated proteins and their role in phenotype.

We hypothesize that PKP2^TR^ is associated with haploinsufficiency, but this can be compensated by the normal allele. On the other hand missense variation may act through a dominant negative effect, disrupting the normal functioning of the wild type protein. Further studies in cellular models will be necessary to understand the role of PKP2^TR^ and missense variations in the pathophysiological ARVC process, but the data indicate that the severity of truncated proteins are not as clear as previously believed. This has important implications for the genetic diagnostic field in structural cardiomyopathies.

In conclusion, this study reports a detailed genetic analysis of desmosomal ARVC-associated genes in a Spanish cohort. Genetic analysis revealed truncating PKP2 mutation as the most frequent ARVC related genetic variation. However, in contrast to previous studies, we show that missense mutations have a worse clinical presentation, pointing towards the possible role of mutations as causing a dominant-negative effect on the normal allele. Future genotype-phenotype studies in larger cohorts will either confirm or refute this observation. However, at present this has important implications for clinical decision making, in which truncated proteins are believed of worse functional severity, and therefore are considered responsible for the pathological phenotype [34].

### Study limitations

We believe that this study provides a general overview of the clinical and genetic profile of an ARVC cohort from Spain. However, some limitations should be noted. First, in any genetic study there is a concern of biased patient selection. Patients included in this study were clinically evaluated after symptom presentation, and age of this clinical diagnosis has been taken as age of ARVC onset. The possible bias due to the selected sample should be taken into account when applying genetic testing for the diagnosis in other populations. A second limitation is the mutation distribution; stop-gain mutations are mostly in *PKP2* gene, while missense mutations are distributed in all genes. More studies are needed to clarify the definitive role of stop-gain mutations. The third limitation is the reduced number of patients due to the low prevalence of the disease. Further genotype-phenotype studies of Spanish cohorts are needed, including a larger number of patients and relatives to support the data obtained. In addition, we cannot discard that patients without identified genetic variation could carry a pathogenic mutation in a known gene with minor prevalence or in an unknown gene, so far, or other genetic defects such as copy number variations (CNV) could be also responsible for negative ARVC cases in our cohort, accordingly to recent published studies [36], [37]. Finally, studies in cellular models were not performed, which would complement these results to fully understand the role of PKP2^TR^ and missense variations in the pathophysiological ARVC process.

## Supporting Information

File S1 **Figure S1.** Conservation of the altered aminoacids in novel mutations. Aminoacids are represented by standard abbreviation. (*) Indicates conserved aminoacid among species. Rectangle indicates the position of the mutation. A- Amino acid alignment for PKP2 p.L687. B- Amino acid alignment for DSP p.A2019. C- Amino acid alignment for DSG2 p.C814. **Figure S2-** Electrocardiogram of index case 12 carrying c.2440 T>C p.C814R variation in *DSG2.* **Figure S3-** Electrocardiogram of index case 16 carrying c. 1162 C>T p.R388W variation in *PKP2* gene. **Figure S4-** Electrocardiogram of index case 8 carrying c.137G>A p.R46Q variation in *DSG2* gene. **Figure S5-** Electrocardiogram of index case 4 carrying c.1237C>T p.R413* variation in *PKP2* gene. **Figure S6-** Electrocardiogram of index case 6 carrying c.1912C>T p.Q638* variation in *PKP2* gene. **Figure S7-** Electrocardiogram of index case 13 carrying c.275T>A p.L92* variation in *PKP2* gene. **Figure S8-** Electrocardiogram of index case 3 carrying c.2203C>G p.R735*variation in *PKP2* gene. **Figure S9-** Electrocardiogram of index case 1 carrying c.2956C>T p.Q986* variation in *DSP* gene. **Figure S10-** Electrocardiogram of index case 2 carrying c.2013delC p.P671Pfs12* variation in *PKP2* gene. **Figure S11.** Electrocardiogram of index case 29 carrying c.2194 T>G p.L732V in *DSC2* gene. **Table S1**. **Comparison table of index cases and statistics results.** Evaluated variables for statistical analysis were Task Force Criteria score (giving two points for major criteria and one point for minor criteria) and age at the diagnosis. **Table S2**. **Comparison table of all genetic carriers and statistics results**. **Table S3. Clinical information of relatives carriers included in the study.** N/S - not shown. N/E - Not evaluated. N/A - Not available.
(DOC)
