# Supplementary material for: Stop-Gain Mutations in PKP2 Are Associated with a Later Age of Onset of Arrhythmogenic Right Ventricular Cardiomyopathy
Source: PLoS One. 2014 Jun 26;9(6):e100560. doi: 10.1371/journal.pone.0100560 (PMC4072667; doi:10.1371/journal.pone.0100560)
Supplement: File S1 — Figure S1. Conservation of the altered aminoacids in novel mutations. Aminoacids are represented by standard abbreviation. (*) Indicates conserved aminoacid among species. Rectangle indicates the position of the mutation. A- Amino acid alignment for PKP2 p.L687. B- Amino acid alignment for DSP p.A2019. C- Amino acid alignment for DSG2 p.C814. Figure S2- Electrocardiogram of index case 12 carrying c.2440 T>C p.C814R variation in DSG2. Figure S3- Electrocardiogram of index case 16 carrying c. 1162 C>T p.R388W variation in PKP2 gene. Figure S4- Electrocardiogram of index case 8 carrying c.137G>A p.R46Q variation in DSG2 gene. Figure S5- Electrocardiogram of index case 4 carrying c.1237C>T p.R413* variation in PKP2 gene. Figure S6- Electrocardiogram of index case 6 carrying c.1912C>T p.Q638* variation in PKP2 gene. Figure S7- Electrocardiogram of index case 13 carrying c.275T>A p.L92* variation in PKP2 gene. Figure S8- Electrocardiogram of index case 3 carrying c.2203C>G p.R735*variation in PKP2 gene. Figure S9- Electrocardiogram of index case 1 carrying c.2956C>T p.Q986* variation in DSP gene. Figure S10- Electrocardiogram of index case 2 carrying c.2013delC p.P671Pfs12* variation in PKP2 gene. Figure S11. Electrocardiogram of index case 29 carrying c.2194 T>G p.L732V in DSC2 gene. Table S1. Comparison table of index cases and statistics results. Evaluated variables for statistical analysis were Task Force Criteria score (giving two points for major criteria and one point for minor criteria) and age at the diagnosis. Table S2. Comparison table of all genetic carriers and statistics results. Table S3. Clinical information of relatives carriers included in the study. N/S - not shown. N/E - Not evaluated. N/A - Not available. (DOC) [file pone.0100560.s001.doc]

Supplemental Material

**
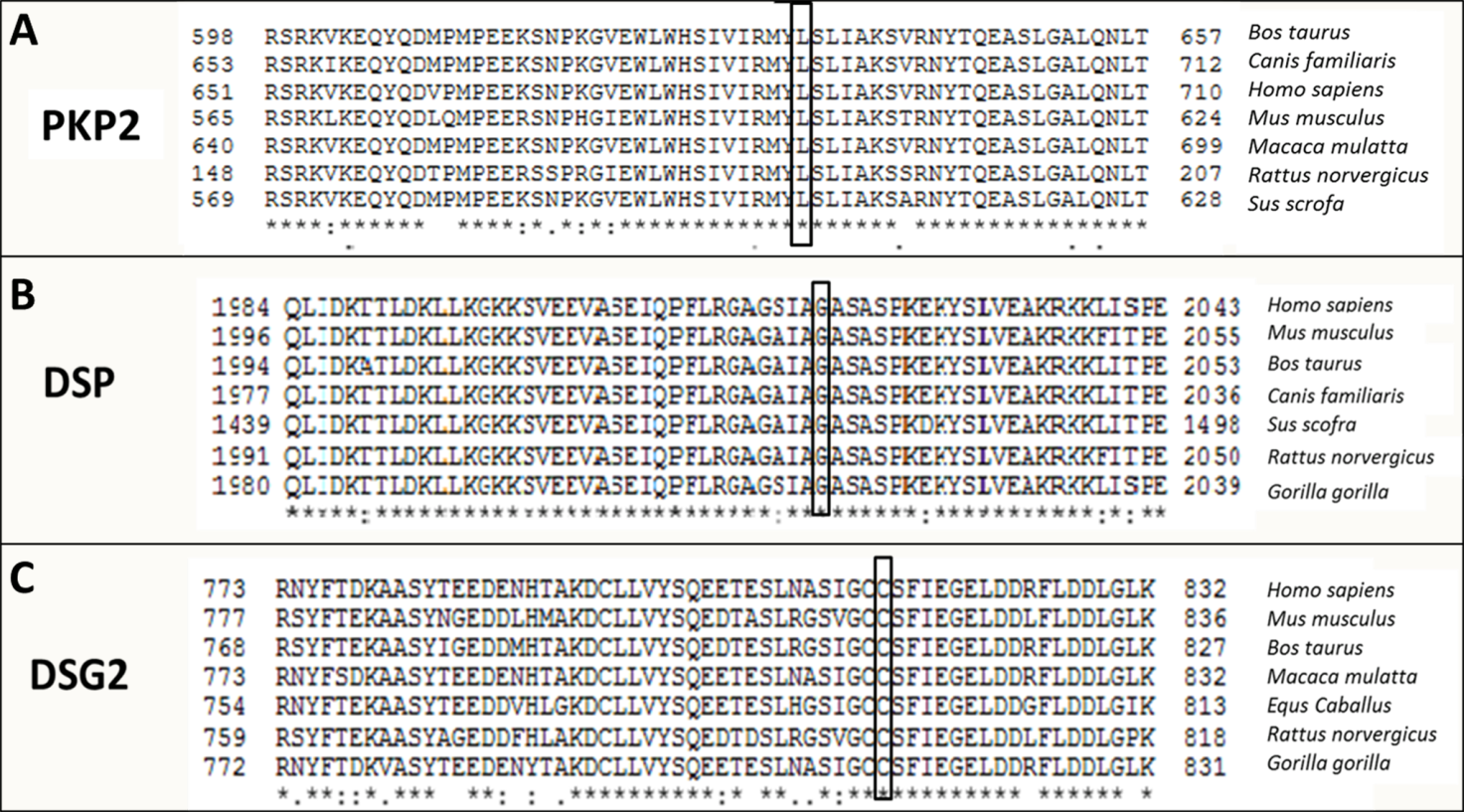
**

**Figure S1.** Conservation of the altered aminoacids in novel mutations. Aminoacids are represented by standard abbreviation. (*) Indicates conserved aminoacid among species. Rectangle indicates the position of the mutation. A- Amino acid alignment for PKP2 p.L687. B- Amino acid alignment for DSP p.A2019. C- Amino acid alignment for DSG2 p.C814.


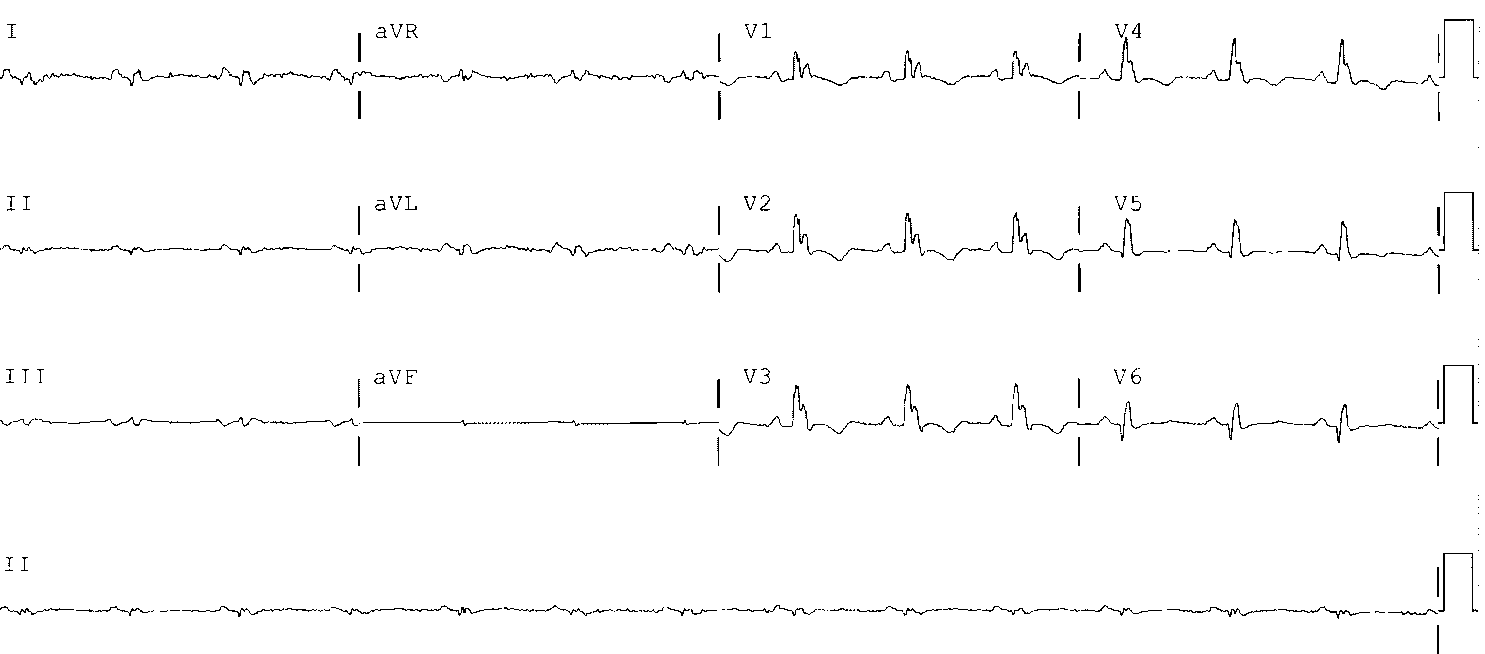


**Figure S2-** Electrocardiogram of index case 12 carrying c.2440 T>C p.C814R variation in *DSG2.*

*
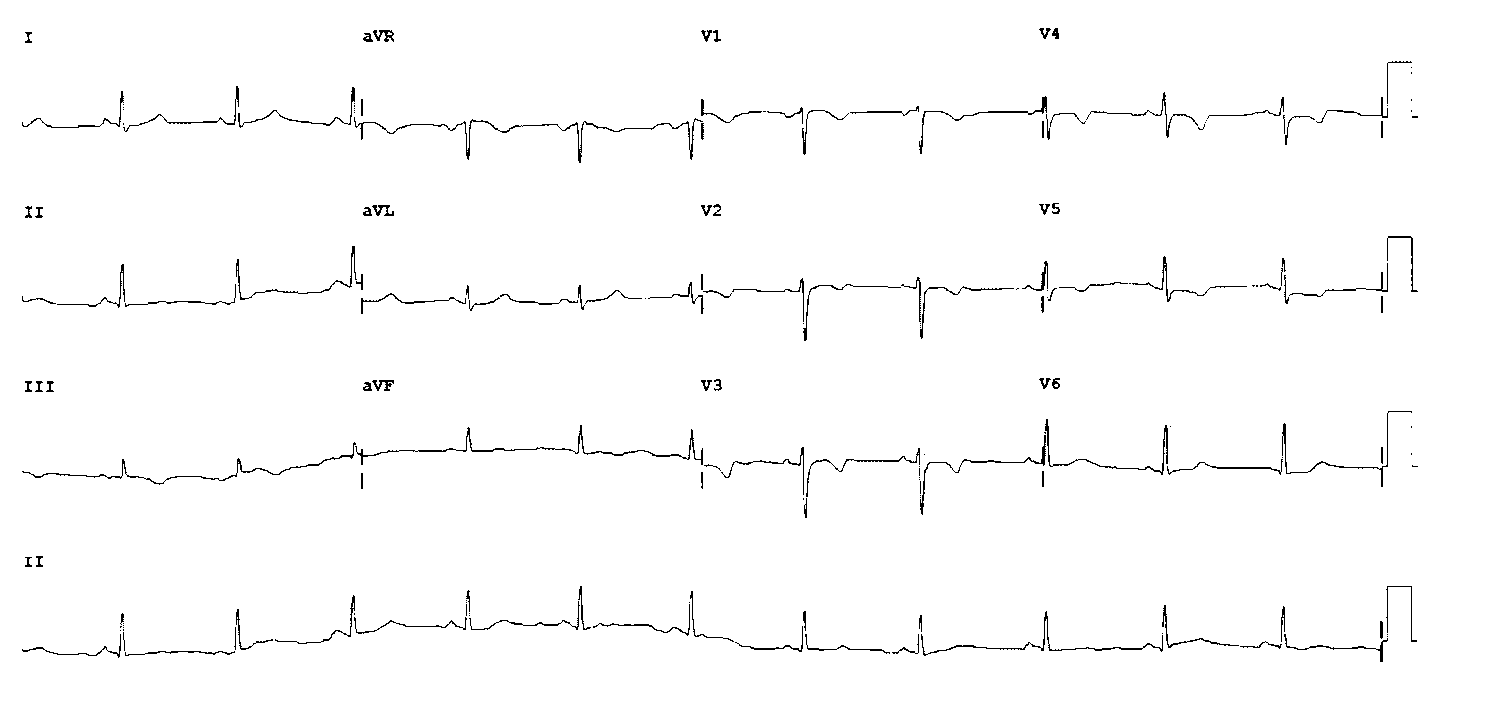
*

**Figure S3-** Electrocardiogram of index case 16 carrying c. 1162 C>T p.R388W variation in *PKP2* gene.


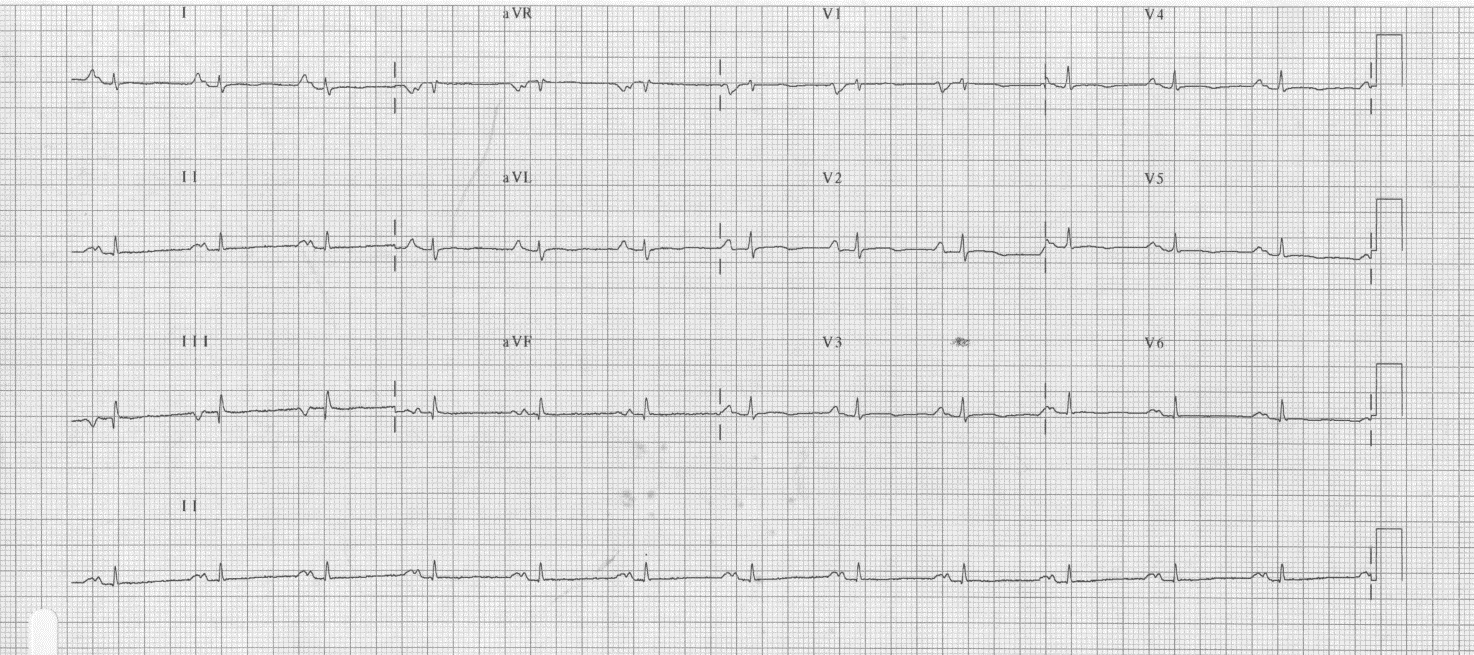


**Figure S4-** Electrocardiogram of index case 8 carrying c.137G>A p.R46Q variation in *DSG2* gene.


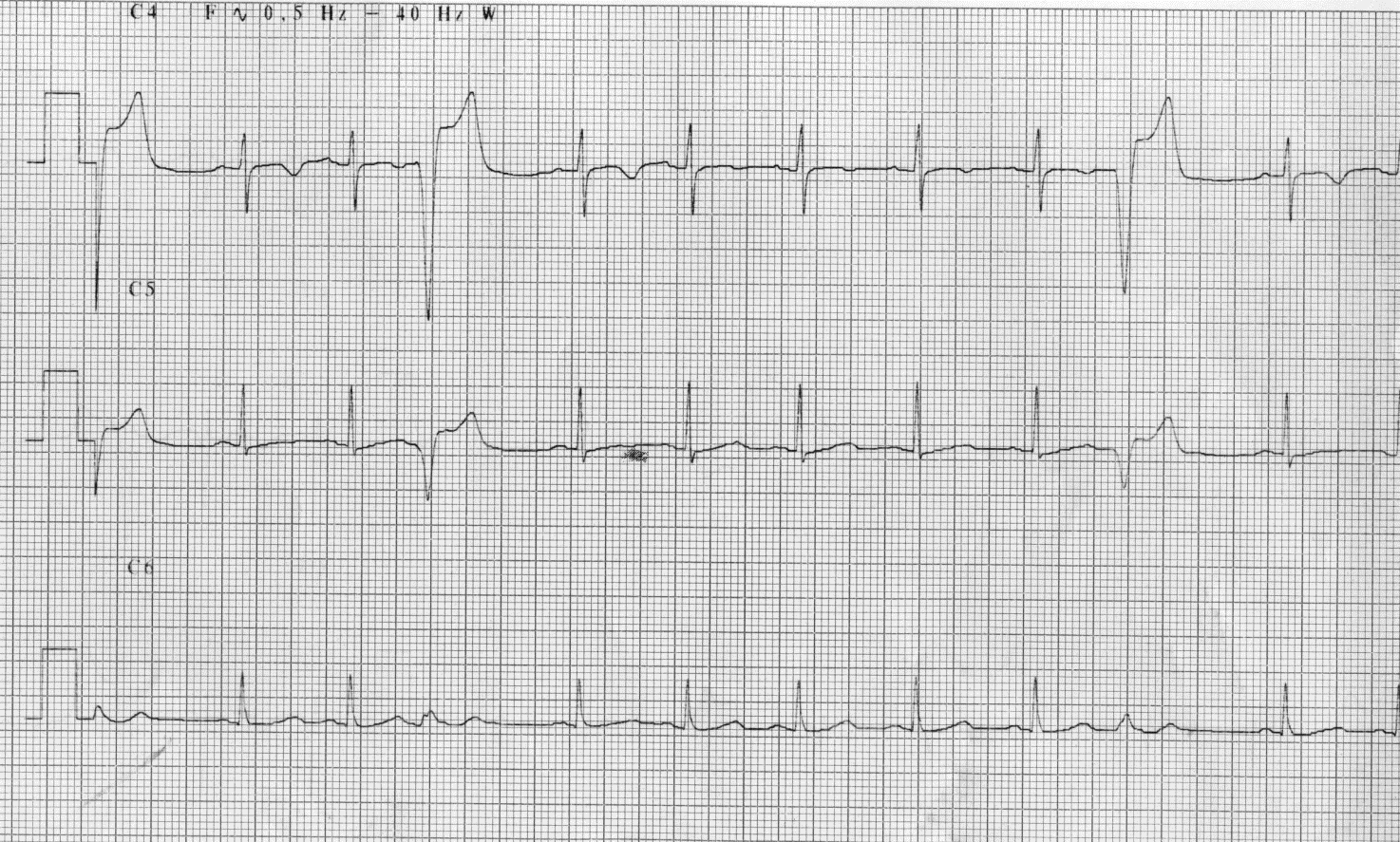


**Figure S5-** Electrocardiogram of index case 4 carrying c.1237C>T p.R413* variation in *PKP2* gene.

**
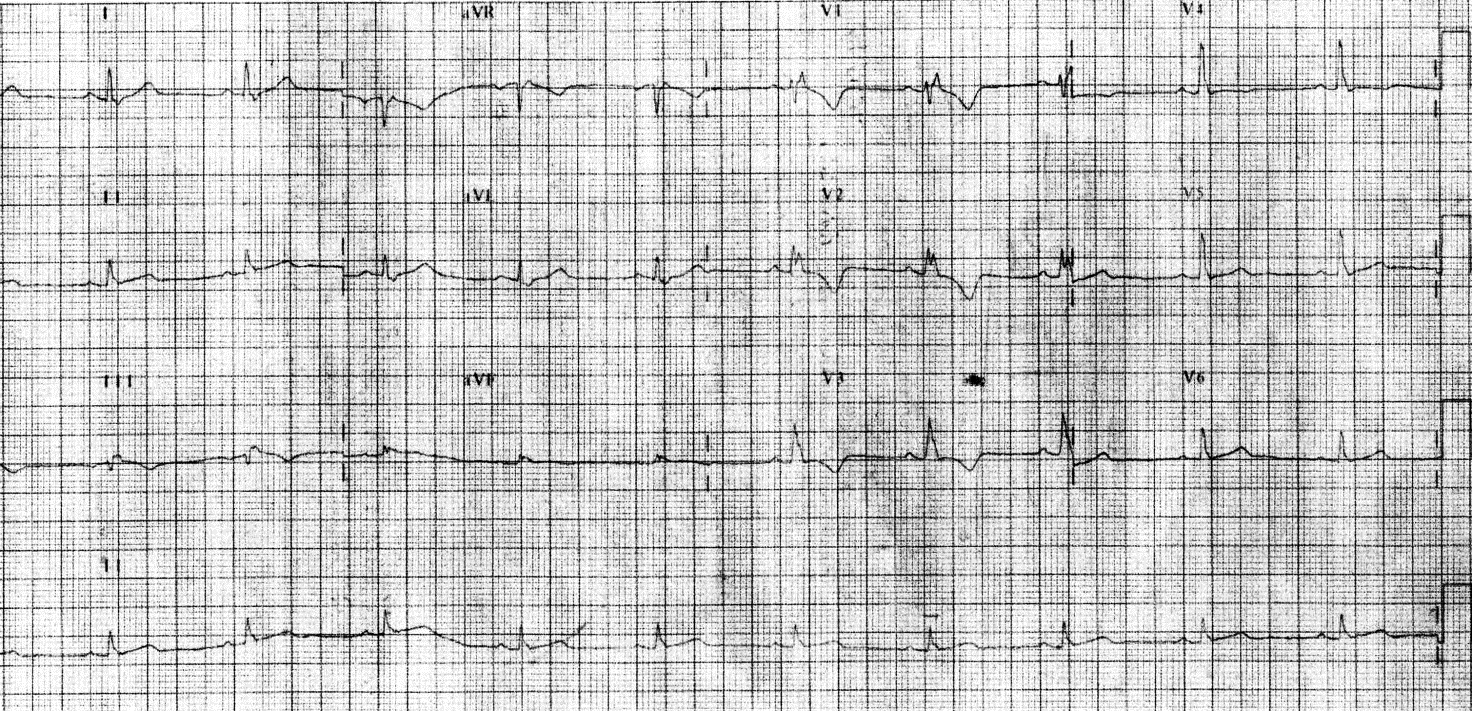
**

**Figure S6-** Electrocardiogram of index case 6 carrying c.1912C>T p.Q638* variation in *PKP2* gene.


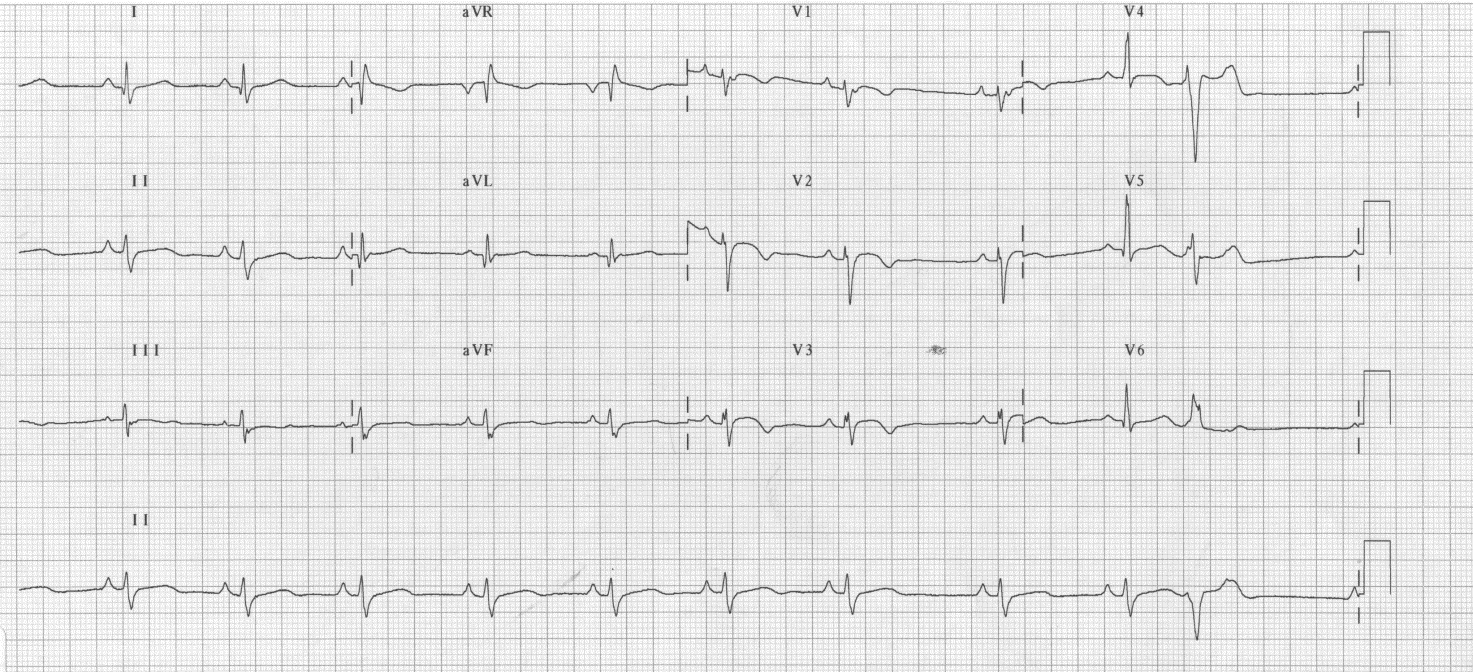


**Figure S7-** Electrocardiogram of index case 13 carrying c.275T>A p.L92* variation in *PKP2* gene.


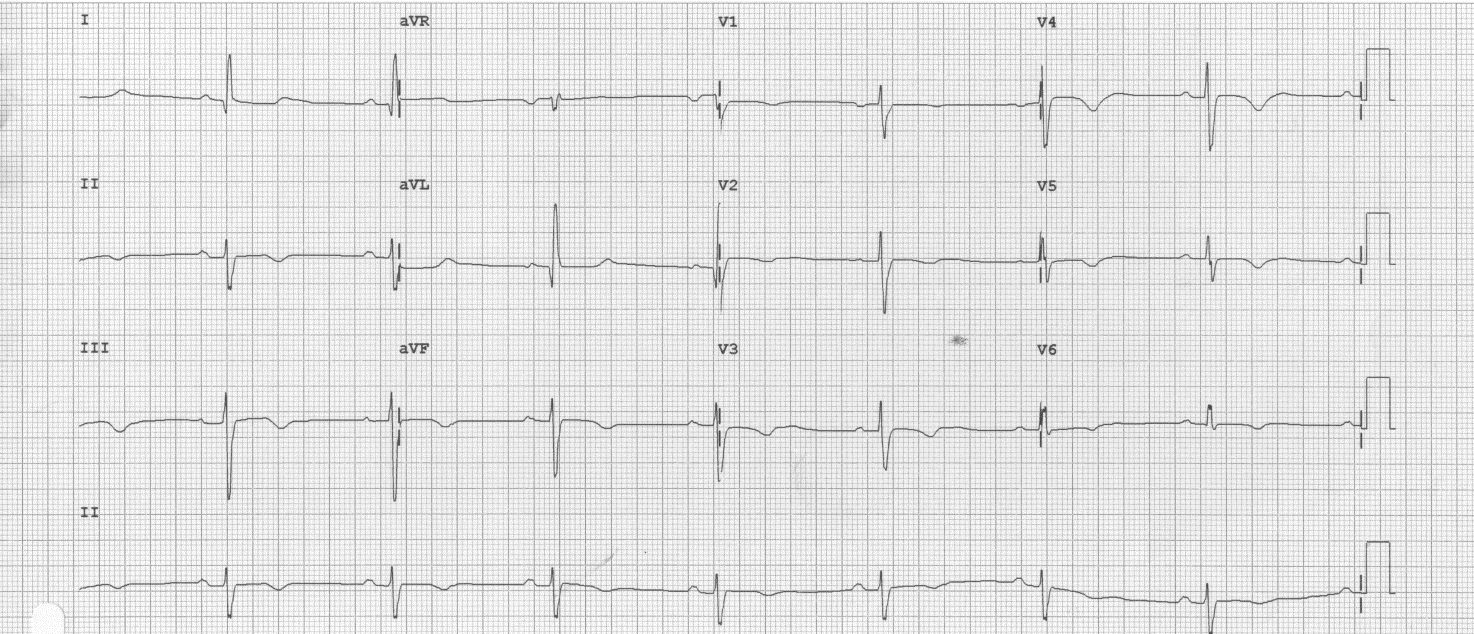


**Figure S8-** Electrocardiogram of index case 3 carrying c.2203C>G p.R735*variation in *PKP2* gene.


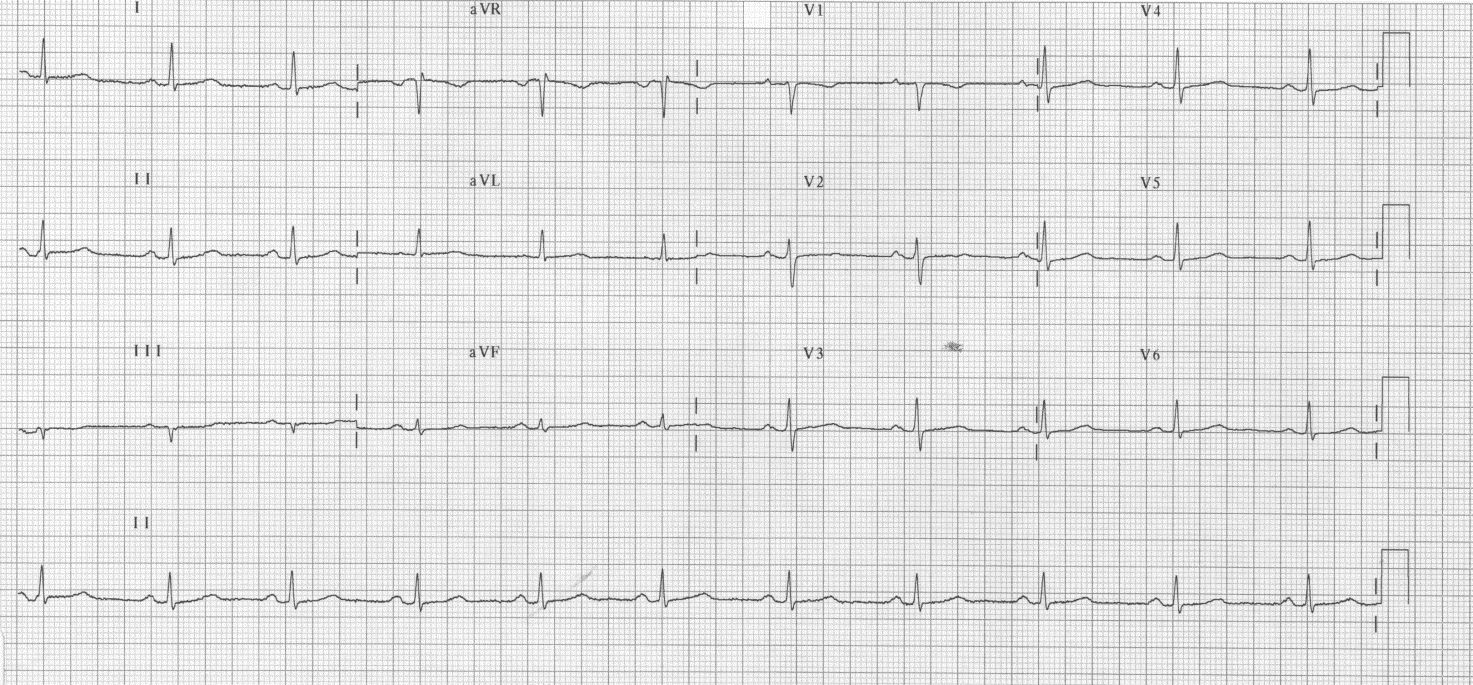


**Figure S9-** Electrocardiogram of index case 1 carrying c.2956C>T p.Q986* variation in *DSP* gene*.*


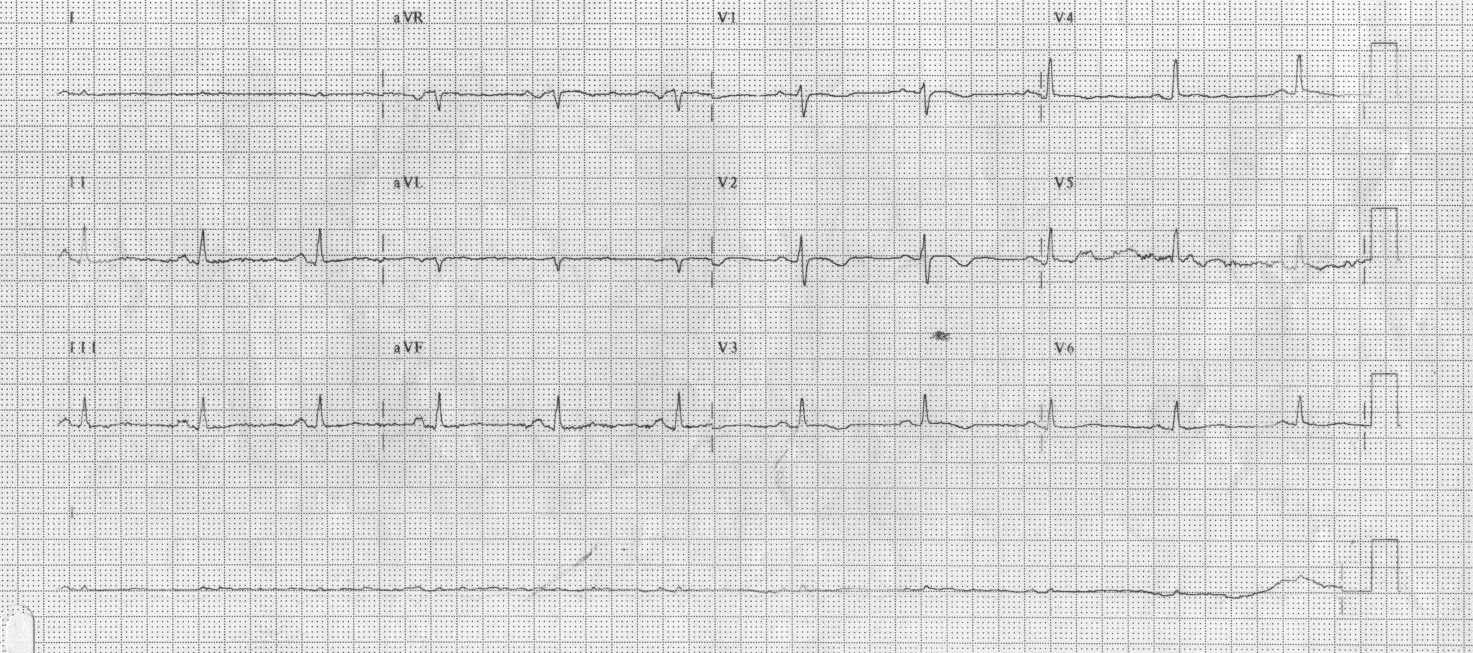


**Figure S10-** Electrocardiogram of index case 2 carrying c.2013delC p.P671Pfs12* variation in *PKP2* gene*.*

*
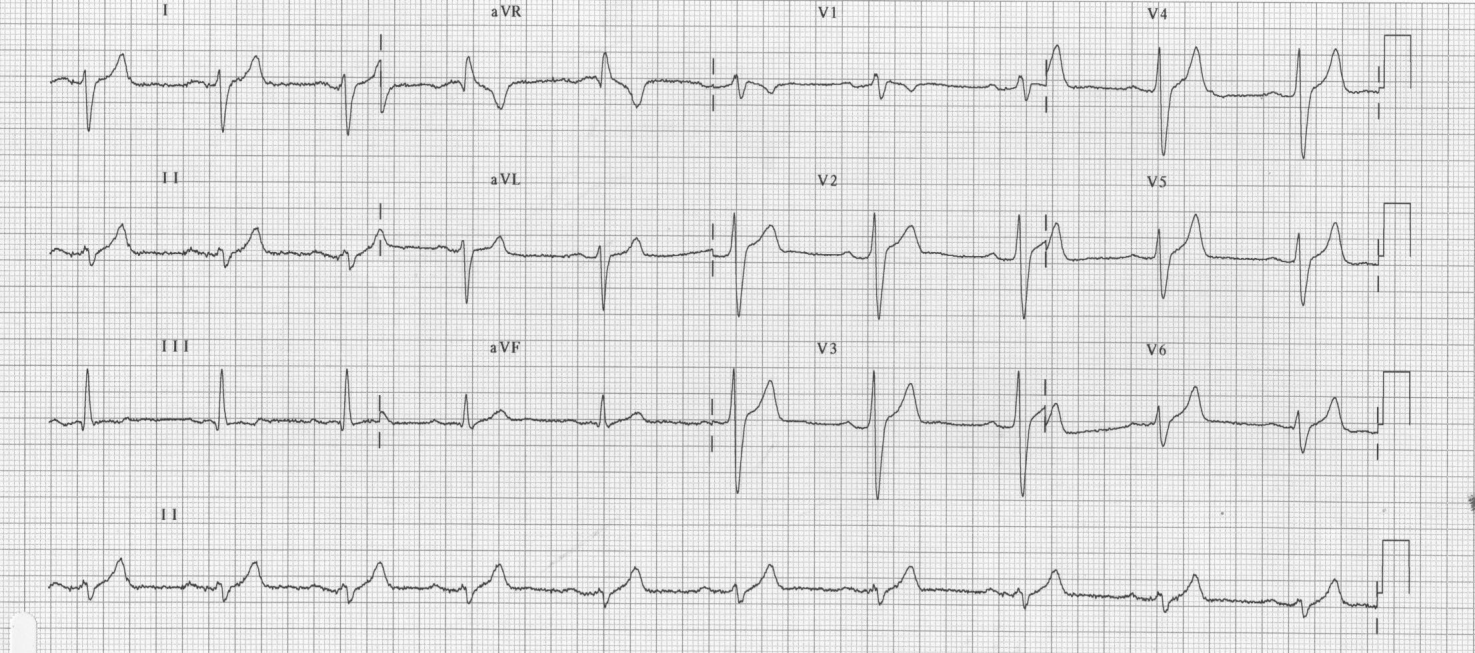
*

**Figure S11.** Electrocardiogram of index case 29 carrying c.2194 T>G p.L732V in *DSC2* gene.

**Table S1**. **Comparison table of index cases and statistics results**.

|  |  |  |  | **Task Force Criteria** |  |  |  |
| --- | --- | --- | --- | --- | --- | --- | --- |
| **Factor** | **Group** | **N** | **Mean** | **Std. Deviation** | **Std. Error Mean** | **Statistical test** | **Sig** |
| **Mutation** | Not identified mutation | 10 | 6,60 | 1,578 | ,499 | T-test | >0.05 |
|  | Identified mutation | 19 | 7,42 | 2,090 | ,479 |
| **Mutation type** | Missense | 8 | 7,38 | 1,685 | ,596 | T-test | >0.05 |
|  | Stop-gained | 11 | 7,45 | 2,423 | ,731 |
| **Affected Gene** | PKP2 | 13 | 7,62 | 2,142 | ,594 | One-Way ANOVA | >0.05 |
|  | DSP | 2 | 6,50 | 3,536 | 2,500 |
|  | DSG2 | 3 | 7,33 | 2,082 | 1,202 |
|  | DSC2 | 1 | 7 | - | - |
| **Gender** | Male | 18 | 7,39 | 2,004 | ,472 | T-test | >0.05 |
|  | Female | 11 | 6,73 | 1,849 | ,557 |

|  |  |  |  | **Age of diagnosis** |  |  |  |
| --- | --- | --- | --- | --- | --- | --- | --- |
| **Factor** | **Group** | **N** | **Mean** | **Std. Deviation** | **Std. Error Mean** | **Statistical test** | **Sig** |
| **Mutation** | Not identified mutation | 11 | 35.73 | 10.734 | 3.236 | T-test | >0.05 |
|  | Not identified mutation | 19 | 34.47 | 13.142 | 3.015 |
| **Mutation type** | Missense | 8 | 27.25 | 10.820 | 3.825 | T-test | **<0.05** |
|  | Stop-gained | 11 | 39.73 | 12.523 | 3.776 |
| **Affected Gene** | PKP2 | 13 | 37.62 | 11.850 | 3.287 | One-Way ANOVA | >0.05 |
|  | DSP | 2 | 30.50 | 27.577 | 19.5 |
|  | DSG2 | 3 | 28.67 | 9.018 | 5.207 |
|  | DSC2 | 1 | 19 | - | - |
| **Gender** | Male | 19 | 33,21 | 12,372 | 2,838 | T-test | >0.05 |
|  | Female | 11 | 37,91 | 11,674 | 3,520 |

Evaluated variables for statistical analysis were Task Force Criteria score (giving two points for major criteria and one point for minor criteria) and age at the diagnosis.

**Table S2**. **Comparison table of all genetic carriers and statistics results**.

|  |  |  |  | **Diagnosis Age** |  |  |  |
| --- | --- | --- | --- | --- | --- | --- | --- |
| **Factor** | **Group** | **N** | **Mean** | **Std. Deviation** | **Std. Error Mean** | **Statistical test** | **Sig** |
| **Mutation type** | Missense | 8 | 27.25 | 10.820 | 3.825 | T-test | **<0.05** |
|  | Stop-gained | 22 | 38.73 | 12.519 | 2.299 |
| **Gender** | Male | 18 | 29,17 | 8,670 | 2,503 | T-test | >0.05 |
|  | Female | 12 | 36.58 | 13,165 | 3,103 |

**Table S3. Clinical information of relatives carriers included in the study.**

| **Family** | **Pedigree position** | **Mutations Genotype** | **ARVC TFC+** | **Age** | **Gender** | **Symptoms** | **I.RV size/function** | **II.RV Histology** | **III. Repolarization** | **IV Depolarization** | **V. Arrhythmias** | **VI. SCD History** | **Diagnostic**  **Score** |
| --- | --- | --- | --- | --- | --- | --- | --- | --- | --- | --- | --- | --- | --- |
| N/S | N/S | DSP p.Q986* | Yes | 15 | F | No | Major | - | - | Minor | - | Major | 5 |
| N/S | N/S | DSP p.Q986* | Yes | 53 | F | Syncope | Minor | - | Major | - | Major | Major | 5 |
| N/S | N/S | DSP p.Q986* | No | 46 | M | No | - | - | - | - | - | Major | 2 |
| N/S | N/S | DSP p.Q986* | No | 42 | F | No | - | - | - | - | - | Major | 2 |
| N/S | N/S | DSP p.Q986* | No | 16 | F | No | - | - | - | - | - | Major | 2 |
| N/S | N/S | DSP p.Q986* | No | 82 | M | No | NE | NE | NE | NE | NE | Major | 2 |
| N/S | N/S | DSP p.Q986* | No | 10 | M | No | - | - | - | - | - | Major | 2 |
| N/S | N/S | DSP p.Q986* | No | 11 | F | No | - | - | - | - | - | Major | 2 |
| B | II.3 | PKP2 p.P671Pfs12* | Yes | 58 | M | Syncope | Major | - | Minor | - | Minor | Major | 6 |
| B | III.3 | PKP2 p.P671Pfs12* | No | 27 | M | No | - | - | - | - | - | Major | 2 |
| A | IV.7 | PKP2 p.R735* | No | 20 | M | No | - | - | - | - | - | Major | 2 |
| A | III.3 | PKP2 p.R735* | Yes | 58 | F | No | Major/Minor | - | Major | - | - | Major | 7 |
| A | IV.5 | PKP2 p.R735* | Yes | 25 | F | No | Major/Minor | - | Major | - | - | Major | 7 |
| A | III.2 | PKP2 p.R735* | Yes | 55 | F | No | Minor | - | Minor | - | - | Major | 4 |
| A | IV.6 | PKP2 p.R735* | No | 36 | F | - | - | - | - | - | - | Major | 2 |
| A | IV.4 | PKP2 p.R735* | No | 27 | F | No | - | - | - | - | - | Major | 2 |
| D | II.4 | PKP2 p.R413* | Yes | 35 | M | No | Major /Minor | - | Minor | - | - | Major | 6 |
| D | III.2 | PKP2 p.R413* | Yes | 15 | M | Palpitations | Major | - | Minor | - | - | Major | 5 |
| D | II.6 | PKP2 p.R413* | Yes | 29 | M | Arrhythmic storm | Major | - | Minor | - | Major | Major | 7 |
| D | II.3 | PKP2 p.R413* | Yes | 16 | M | Sudden death | N/A | N/A | N/A | N/A | N/A | - | - |
| C | I.2 | PKP2 p.Q638* | No | 80 | M | No | - | - | - | - | - | Major | 2 |
| C | II.2 | PKP2 p.Q638* | No | 41 | F | No | - | - | - | - | - | Major | 2 |
| C | III.1 | PKP2 p.Q638* | No | 9 | M | No | - | - | - | - | - | Major | 2 |
| C | II.6 | PKP2 p.Q638* | Yes | 35 | F | No | Minor | - | Major/Minor | - | - | Major | 6 |
| E | II.5 | DSG2  p.C814R | No | - | M | No | - | - | - | - | - | Major | 2 |
| E | II.6 | DSG2  p.C814R | No | - | F | No | - | - | - | - | - | Major | 2 |
| E | III.1 | DSG2  p.C814R | No | - | F | No | - | - | - | - | - | Major | 2 |
| N/S | N/S | DSG2 p.R46Q | No | 64 | F | No | - | - | - | - | - | Major | 2 |
| N/S | N/S | PKP2 p.L92* | No | 40 | F | No | - | - | - | - | - | Major | 2 |
| N/S | N/S | PKP2 p.R388W | No | 64 | M | No | - | - | - | - | - | Major | 2 |
| N/S | N/S | DSC2 p.L732V | No | 60 | M | No | - | - | - | - | - | Major | 2 |

N/S- not shown. N/E-Not evaluated. N/A- Not available
